# Preserving Vitality: A Case Report of Partial Pulpotomy in Dental Practice

**DOI:** 10.7759/cureus.61720

**Published:** 2024-06-05

**Authors:** Saee Wazurkar, Aditya Patel, Manoj Chandak, Anuja Ikhar, Namrata Jidewar, Lalit Pawar, Mrinal Nadgouda

**Affiliations:** 1 Department of Conservative Dentistry and Endodontics, Sharad Pawar Dental College and Hospital, Datta Meghe Institute of Higher Education and Research, Wardha, IND

**Keywords:** partial pulpotomy, bioceramic material, pulpotomy, mineral trioxide aggregate (mta), vital pulp therapy

## Abstract

This paper aims to evaluate the outcomes of a partial pulpotomy with mineral trioxide aggregate (MTA) in a maxillary first premolar with reversible pulpitis symptoms and signs. An intraoral periapical radiograph revealed a deep pulp-involving carious lesion without any indications of a periapical lesion, no history of night pain, and no tooth tenderness when percussion was applied. Caries removal is done using a round bur, 2-3 mm of inflamed pulp from the crown portion was removed, and bleeding was controlled within four minutes using 2.5% sodium hypochlorite, over which MTA was placed. After the setting of MTA, resin-modified glass ionomer cement was placed over it, and the tooth was restored using composite. The patient was asymptomatic in six months and one-year follow-up with no periapical changes and showed dentin bridge formation. Careful case selection, a precise selection of biomimetic material, and long-term follow-up validate the success of the treatment.

## Introduction

It's essential to avoid irreversible dental procedures like root canal treatment or extractions whenever we can, especially when dealing with young patients [1]. With the advancement of biomimetic material, minimally invasive dental procedures are possible even in caries reaching nearer to the pulp or involving the pulp, depending on the signs and symptoms one can decide on a vital therapy [2].

In 2015, recommendations were made at the International Caries Consensus Collaboration conference in Leuven, Belgium, indicating that maintaining pulp vitality should be the primary focus when managing severe carious lesions [3]. But even after a careful and conservative approach is followed, sometimes pulp exposure is inevitable. In such cases, endodontic treatment should be performed [4]. In dental literature, Cvek first used "partial pulpotomy" in 1978. Many authors observed a significant recovery rate of 96% when 2-3 mm of inflamed pulp tissue was surgically amputated from teeth with crown fractures [2]. Partial pulpotomy can be performed in cases of cuspal fracture, deep dentinal caries, or damage due to enamel developmental defects [5].

Partial pulpotomy differs from cervical pulpotomy as it includes the removal of 2-3 mm from the coronal pulp tissue, unlike cervical pulpotomy, which consists of removing the whole coronal portion of the dental pulp [6]. Partial pulpotomy is preferred over coronal pulpotomy as the remaining viable coronal tissue helps in exaggerated healing of the remaining pulp, enhancing the long-term prognosis of the tooth [7]. According to the American Association of Endodontics, complete caries removal is important. Residual caries may contain infectious tissue and visualize the condition of pulp tissue under magnification [8]. More invasive therapy might be needed if hemostasis is unable to be achieved. Hemostasis should be achieved within four to 10 minutes for healthy pulp; however, different time frames are stated in the literature [9].

With newly developed materials and techniques offering enhanced biocompatibility and a robust seal, a partial pulpotomy can be performed more confidently [10]. For the past 10 years, mineral trioxide aggregate (MTA) has been used as a pulp capping agent due to its biocompatibility, ability to form a dentinal bridge, sealing ability, and ability to maintain high pH for longer periods. Earlier calcium hydroxide has been used as a pulp capping agent, but due to its toxicity, inability to adhere to dentin, and degradation over time led to tunnel defects, due to its shortcomings calcium hydroxide lacks popularity [11,12].

Another newly developed silicate-based material, Biodentine (Septodont, Saint-Maur-des-Fossés, France) shows excellent biocompatibility with accelerated tissue regeneration properties and the ability to stimulate dentin as this material has reduced setting time [13]. As there are fewer clinical trials depicting the outcomes of Biodentine, MTA was selected as the material of choice for the study [14]. This case report aims to present a successful outcome of partial pulpotomy, in which 2-3 mm of inflamed coronal pulp is removed to maintain pulp vitality, using biomimetic material like MTA and restored using resin composite. The successful outcome of this procedure depends on factors such as age, bleeding time, and extent of the carious lesion.

## Case presentation

A 21-year-old female reported to Sharad Pawar Dental College and Hospital in Wardha, India, with a chief complaint of pain and sensitivity to cold, which was relieved after the removal of stimulus but currently had no pain. The medical history was insignificant. On clinical examination, proximal caries were found with tooth number 15, which was non-tender on percussion. There was no history of night pain, sinus discharge, or associated fever. Electrical pulp testing showed an early response compared to the adjacent and contralateral tooth on the cold test, which was performed using Endo ice refrigerant spray (Coltene, Cuyahoga Falls, USA) and showed no sign of pain after removal of stimulus.

**Figure 1:**
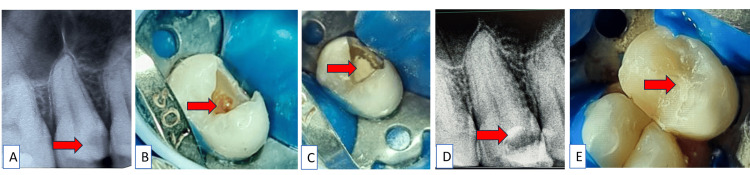
A) Pre-operative radiograph shows carious tooth involving pulp with tooth number 15; B) Caries excavation and hemostasis established using a cotton pellet soaked in NaOCl for two minutes; C) Clinical photograph of MTA placement; D) Periapical radiograph of placement of MTA with tooth number 15; E) Composite restoration done after MTA placement with tooth number 15 MTA: mineral trioxide aggregate

According to the Federation Dentaire Internationale (FDI) tooth notation system, with tooth number 15, the radiographic evaluation showed deep proximal caries involving the coronal pulp, with no signs of periapical radiolucency and intact lamina dura (Figure 1A). After correlating clinical and radiological signs, we diagnosed the condition as reversible pulpitis with tooth number 15. The whole procedure, including both root canal treatment and vital pulp therapy (VPT), was explained to the patient. If objective and subjective signs of VPT were not achieved, the procedure could also lead to root canal treatment, and informed consent was taken from the patient. Local anesthesia (LA) and rubber dam isolation were done after achieving the signs of LA, caries were excavated using a sterile round bur (BR 41; MANI, Tochigi, Japan) after pulpal exposure 2-3 mm was amputated using a high-speed round bur. After rinsing the cavity with a 2.5% sodium hypochlorite (NaOCl) solution, the pulp tissues were examined at a 3x magnification using the pulpotomy level (Dental Loupe; Eighteeth, Changzhou, China). Applying a 2.5% NaOCl-soaked cotton pellet, bleeding was controlled within four minutes (Figure 1B). Pulp tissue that is pinkish-reddish is regarded as normal. Next, the MTA Angelus (Angelus, Londrina, Brazil) was condensed over the exposed pulpal tissue (Figure 1C). Since the setting time of MTA Angelus was only 15 minutes (Figure 1D), the entire procedure was completed on the same day. After the setting of MTA, a layer of resin-modified glass-ionomer cement (RMGIC; GC Fuji II LC^®^, Tokyo, Japan) was applied. Next, a sectional metal matrix (Palodent system standard matrices; Dentsply Sirona, Charlotte, USA) was used to create a proximal wall. Over this, composite resin (Tetric-Ceram; Ivoclar Vivadent Inc., Amherst, USA) was applied (Figure 1E).

**Figure 2:**
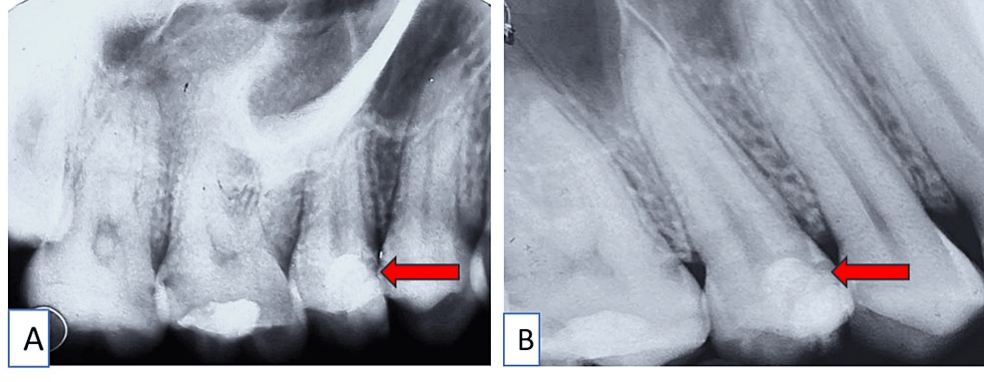
A) Immediate intraoral post-operative radiograph with tooth number 15; B) One-year follow-up radiograph with tooth number 15 The one-year follow-up shows dentin bridge formation and no signs of periodontal ligament widening

An immediate postoperative radiograph was taken (Figure 2A), and the patient was recalled for subsequent follow-up. The patient was satisfied with the treatment, reporting no signs of pain or discomfort during the one-year follow-up. Additionally, no periapical changes or widening of the periodontal ligament were observed, and dentin bridge formation was appreciated (Figure 2B).

## Discussion

Partial pulpotomy is defined as the removal of 2-3 mm of coronal inflamed pulpal tissue and the placement of biomimetic material to maintain the vitality of the pulp [15]. In pulp exposure cases, clinical criteria frequently used to assess the pulp's vitality include the pulp's appearance, the color of the bleeding, and the control of hemorrhage at the exposure site. In this case report, the exposed pulp was vital due to its strong texture, bright red color, and noticeable bleeding from the exposure site [16].

After pulpal amputation, hemorrhage was controlled within four minutes; thus, the pulp was considered to be healthy, and hence, partial pulpotomy was planned. The pulp wound and cavity were cleaned, and hemostasis was achieved in this case reports using 2.5% NaOCl solution. NaOCl has unique characteristics, such as strong antibacterial activity and tissue-dissolving capabilities for soft tissue that has become necrotic. Note that while removing deep caries, if there is pulp exposure and if bleeding is controlled within four minutes, Anta et al. [17] concluded that the time of hemostasis to be five minutes but found that the bleeding stoppage time did not influence the outcome of treatment. According to Baranwal et al. findings, achieving hemostasis typically requires eight to 10 minutes in the majority of cases [16]. If bleeding is not achieved, more pulpal tissue is removed, and the procedure is done again. This may lead to cervical pulpotomy, and when this is not achieved, then a pulpectomy is done [18].

Clinical research has not discovered any significant association between blood clotting times (within one to 10 minutes) and the result of VPT. However, hemostasis achievement is essential for the success of VPT [19]. Therefore, if bleeding persists after attempts to achieve hemostasis, the treatment plan should be modified, going from partial pulpotomy to endodontic treatment [20]. The choice of material is yet another crucial factor in effective VPTs. The gold standard for pulp capping in the past was calcium hydroxide, but clinical trials on VPT for pulps with carious exposure treated with calcium hydroxide have yielded poor outcomes [21].

Therefore, hydraulic calcium silicate-based materials like MTA and Biodentine have demonstrated better clinical and radiographic outcomes compared to calcium hydroxide. Following VPTs, these materials lead to a harder tissue barrier that is both thicker and less porous than calcium hydroxide [22]. Many calcium silicate base materials are available, like MTA and Biodentine. These materials are shown to induce and stimulate the growth factors and odontoblast-like tissue [23].

Elmsmari et al. conducted a systematic review, and they found that patient age and tooth maturity did not affect the outcome of partial pulpotomy, although patients included in this review ranged in age from six to 52 years. Partial pulpotomies have an increased success rate throughout the various re-evaluation periods, despite the lack of a set time frame for when they can be considered successful [20]. According to Matsuo et al., there is no discernible difference in the provisional prognosis of these pulp cappings between three and 18 months. So, 21 months is the appropriate time frame for assessing the likelihood of a direct pulp capping's success [23]. Chailertvanitkul et al. conducted a study to evaluate the treatment outcome of MTA and calcium hydroxide for partial pulpotomy. Within a limited two-year follow-up, there was no significant difference between ProRoot MTA and Dycal. However, cases with large pulp exposure areas (>5 mm^2^) showed less favorable outcomes [2]. Another study, conducted by Bakhtiar et al., aimed to compare the efficacy of TheraCal, Biodentine, and MTA for partial pulpotomy. The results showed that Biodentine and MTA outperformed TheraCal. Incomplete bridge formation was observed with TheraCal due to the microleakage of the monomer, as it is a resin-based material [24].

The final sealing of the restoration plays a crucial role in determining the long-term prognosis of the restoration. If the seal is broken, bacteria may infiltrate the pulp through the mineralized bridge [25]. Thus, the final aim of the VPT should be the formation of a dentine bridge and preserving the vitality of the pulp.

## Conclusions

Pulpotomy is mainly of two types while performed in adult mature teeth: cervical pulpotomy and partial pulpotomy. In this case, partial pulpotomy was performed, and it is concluded that MTA Angelus might be a suitable biomimetic material for the partial pulpotomy of permanent upper premolars. In this case report, 2-3 mm of coronal pulp is removed, and after achieving hemostasis, MTA was placed over the exposed pulp of the tooth, indicating reversible pulpitis. The tooth was restored using resin composite, and the patient was kept on follow-up. After one year of follow-up, there was no pain or sensitivity associated with the tooth, and the radiograph showed no sign of periodontal widening. The formation of the dentinal bridge describes the positive outcome of the study.
